# A 10-Year Comprehensive, Single-Center, Retrospective Analysis on Juxtapleural Nodules: Insights into Classification and Risk

**DOI:** 10.3390/diagnostics16111663

**Published:** 2026-05-28

**Authors:** Isabelle C. Pappas, Quincy A. Hathaway, Hubert A. Gbate, Yashbir Singh, Elena Ghotbi, Friedrich D. Knollmann, Eduardo Mortani Barbosa, Achala Donuru, Dongming Xu

**Affiliations:** 1 Department of Radiology, University of Pennsylvania, Philadelphia, PA 19104, USA; hubert.gbate@pennmedicine.upenn.edu (H.A.G.); friedrich.knollmann@pennmedicine.upenn.edu (F.D.K.); eduardo.barbosa@pennmedicine.upenn.edu (E.M.B.J.); achala.donuru@pennmedicine.upenn.edu (A.D.); 2 Cooper Medical School, Rowan University, Camden, NJ 08103, USA; 3 Department of Radiology, Korle-Bu Teaching Hospital, Accra P.O. Box 77, Ghana; 4 Department of Radiology, Mayo Clinic, Rochester, MN 55905, USA; yashbir143@gmail.com; 5 Department of Radiology and Radiologic Sciences, Johns Hopkins University, Baltimore, MD 21287, USA; eghotbi1@jh.edu

**Keywords:** juxtapleural nodules, perifissural nodules, intrapulmonary lymph nodes, malignancy, thoracic imaging, retrospective study

## Abstract

**Background/Objectives:** Juxtapleural and perifissural nodules are commonly encountered on thoracic imaging, are typically presumed benign, and are often considered to represent intrapulmonary lymph nodes. We evaluated the prevalence, clinical context, and management of juxtapleural nodules over a 10-year period and to assess factors associated with concern for malignancy. **Methods:** In this retrospective, single-center study, radiology reports from 1 January 2015 to 31 December 2024, were queried using natural language processing to identify examinations containing terminology consistent with juxtapleural nodules. After exclusion of duplicate encounters, 85,435 unique patient encounters were analyzed. Reports were further filtered to identify cases with imaging concern for malignancy and recommendations for follow-up or intervention (N = 659). Clinical characteristics, imaging modality, and prior malignancy history were compared using appropriate statistical tests. **Results:** Among 85,435 unique encounters, 659 patients (<1%) had juxtapleural nodules considered concerning for malignancy. These patients were older (68 vs. 64 years, *p* < 0.001), more frequently imaged with PET/CT (8% vs. 5%, *p* < 0.001), and more likely to be evaluated in inpatient settings (16% vs. 12%, *p* < 0.001). Of the cohort with a juxtapleural nodule considered concerning for malignancy by imaging criteria, 360 patients (55%) had a prior history of malignancy. In these patients, nodules were more commonly identified in outpatient settings (70% vs. 43%, *p* < 0.001) and on routine imaging (94% vs. 86%, *p* < 0.001). Representative cases demonstrated that malignant nodules may mimic benign perifissural morphology on initial imaging. **Conclusions:** Although the vast majority of reported juxtapleural nodules did not prompt malignancy-directed evaluation, a small subset of cases (approximately 0.8%), particularly in patients with prior malignancy, were interpreted as sufficiently concerning to warrant follow-up imaging or intervention. Because pathologic confirmation and standardized long-term outcome adjudication were not available for all patients, these findings should be interpreted as reflecting real-world radiologist concern and management patterns rather than a definitive malignancy rate.

## 1. Introduction

Pulmonary nodules are commonly encountered on thoracic imaging performed for a wide range of clinical indications, including cancer surveillance, staging, infection, trauma, cardiopulmonary symptoms, and routine follow-up [1,2]. Although many are ultimately benign, initial classification can be challenging, especially when nodules are pleural-based, fissural, or identified in patients with a prior history of malignancy [1,2,3,4]. The widespread use of CT imaging, including low-dose computed tomography (LDCT) screening after the National Lung Screening Trial (NLST), has further increased the detection of incidental pulmonary nodules in routine practice [4,5].

Among these findings, juxtapleural and perifissural nodules are commonly described in both screening and routine clinical imaging. These nodules are often presumed to represent intrapulmonary lymph nodes (IPLNs) if small (<10 mm), solid, smoothly marginated, and located adjacent to pleural surfaces or fissures. Classic morphologic descriptions include oval, triangular, or lentiform shapes, sometimes with a thin pleural tag extending to the pleural surface, representing ecstatic lymphatic channels or interlobular septa [6,7,8]. In lung cancer screening populations, nodules with imaging characteristics consistent with IPLNs may account for a substantial proportion of non-calcified nodules [9]. Current management guidelines, including the 2017 Fleischner Society recommendations and Lung-RADS v2022, generally consider such nodules benign or probably benign when typical features are present, often recommending routine or annual follow-up rather than short-interval imaging [4,7].

However, nodules initially resembling intrapulmonary lymph nodes may occasionally raise concern for malignancy, particularly in patients with a prior history of cancer or known metastatic disease [10]. In addition, pleural-based and juxtafissural nodules can represent a range of entities beyond IPLNs, including metastatic disease, early primary lung cancer, focal atelectasis, pleural plaques, and inflammatory processes [4,11]. In day-to-day practice, distinguishing between these possibilities is not always straightforward, especially when morphologic features are subtle or when clinical history introduces additional complexity. Overcalling benign nodules may lead to unnecessary imaging or invasive procedures, whereas under-recognition of malignant lesions can delay diagnosis. Despite how frequently juxtapleural nodules are mentioned in radiology reports, relatively little data exist examining their real-world clinical trajectory outside structured screening cohorts. It is also unclear how often these nodules prompt short-term follow-up or malignancy-directed evaluation in routine practice, and whether management differs meaningfully in patients with and without a known history of cancer.

To better understand this, we performed a 10-year retrospective, single-center analysis of radiology reports referencing juxtapleural or perifissural nodules. Our goals were to describe how often these nodules are reported, how frequently they prompt follow-up or further evaluation for malignancy, and how prior cancer history may influence management decisions. By examining a large, unselected cohort across a decade of imaging practice, we aim to provide descriptive data that may help inform more nuanced risk assessment of juxtapleural nodules encountered in clinical practice.

## 2. Methods

### 2.1. Study Design and Cohort Identification

This retrospective study was approved by the institutional review board with waiver of informed consent. All radiologic examinations performed within the Penn Medicine health system between 1 January 2015 and 31 December 2024 were queried using a natural language processing (NLP)-based text search of radiology reports. Patients were initially identified if the radiology report contained the following terms (as well as any derivative versions or alternative spellings):

(1) juxtapleural, subpleural, juxtafissural, perifissural, costal pleural, perimediastinal, or peridiaphragmatic

AND

(2) nodule.

The above search criteria resulted in 174,700 reports. To only include unique patient encounters, any subsequent follow-up visits by a patient (N = 82,285) and duplicate accession numbers (N = 6980) were removed; this resulted in 85,435 unique patient encounters that included a juxtapleural nodule.

To enrich for patients with concern for malignancy, reports were further filtered to include the following:

(1) The Impression section must contain one of the above anatomic descriptors of a juxtapleural nodule

AND

(2) The Impression section was required to include terminology referencing a juxtapleural *nodule* in conjunction with language suggestive of malignancy (i.e., cancer, metastasis, malignancy, or tumor).

**Figure 1:**
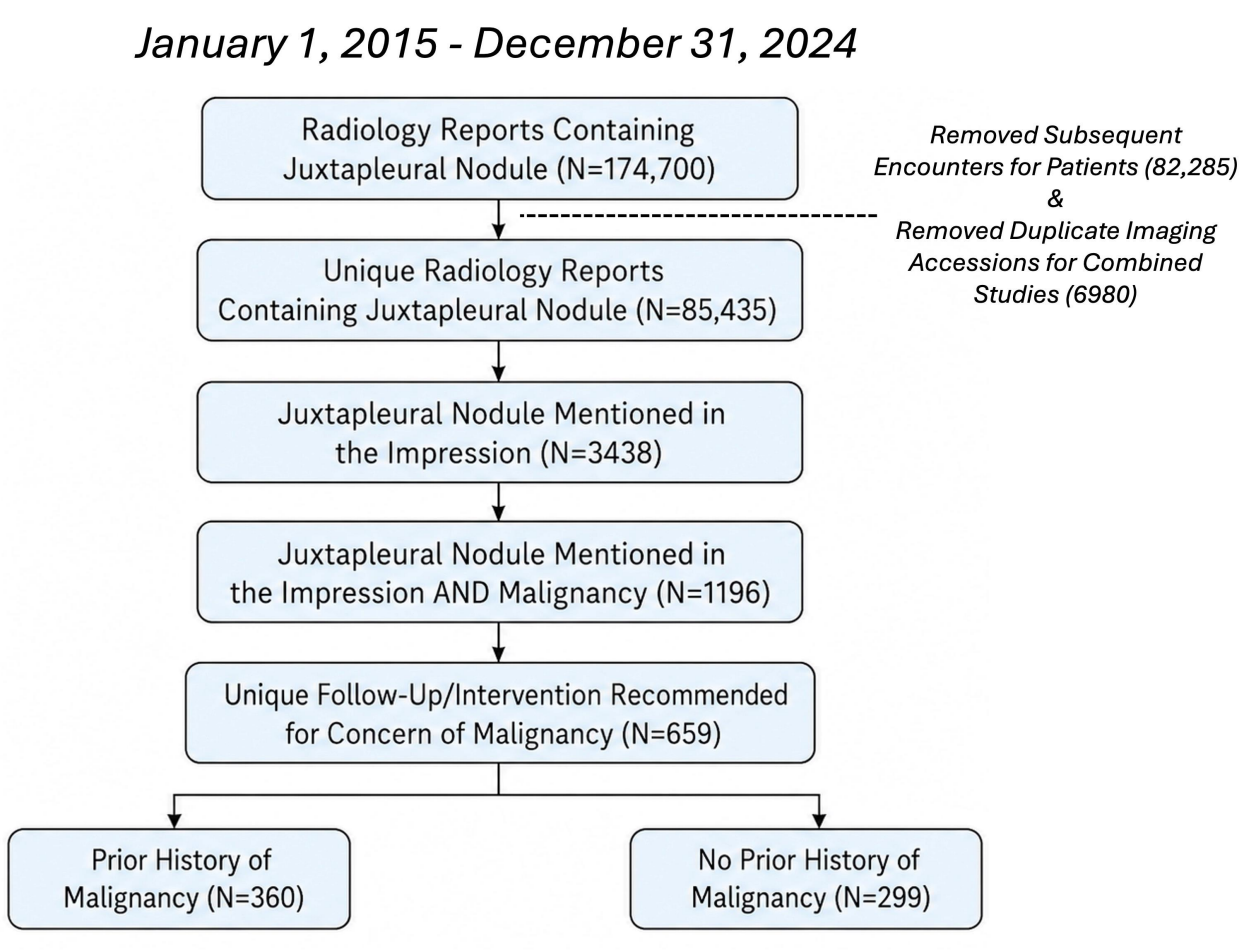
Patient selection flow diagram. Flow diagram illustrating cohort selection from 174,700 radiology reports containing terminology consistent with juxtapleural nodules (1 January 2015–31 December 2024). Sequential exclusions for duplicate encounters and imaging accessions resulted in 85,435 unique patient encounters. Further filtering based on impression-level concern for malignancy and follow-up recommendations yielded the final cohort of 659 patients. Of the 659 patients, 360 patients had a prior reported history of malignancy, and 299 had no reported history of malignancy.

This resulted in 1196 patients eligible for further analysis. Among these 1196 patients, we further excluded patients if there was (1) no clear recommendation for imaging follow-up or intervention and/or (2) they had multiple juxtapleural nodules in the setting of malignancy (N = 537 removed). The final cohort included 659 patients with a juxtapleural nodule that was concerning for malignancy by imaging criteria and close follow-up or intervention (intervention/surgery) was recommended. Clinical history of malignancy was determined through structured data fields within the radiology report by (I.C.P., medical student) with a second review by (Q.A.H, radiology resident). Manual chart review was performed as necessary for cases with limited clinical context in the radiology report. Patients were categorized into those with (N = 360) or without (N = 299) a reported history of malignancy (Figure 1).

The primary endpoint of this study was report-level concern for malignancy resulting in recommendation for follow-up imaging, PET/CT, biopsy, surgery, or other intervention. This endpoint was intentionally defined as a real-world radiology report and management endpoint rather than a pathologically confirmed malignancy endpoint. Pathologic confirmation and standardized longitudinal outcome adjudication were not available for all patients; therefore, the study does not estimate the true malignancy rate of juxtapleural nodules.

### 2.2. Nodule Classification

**Figure 2:**
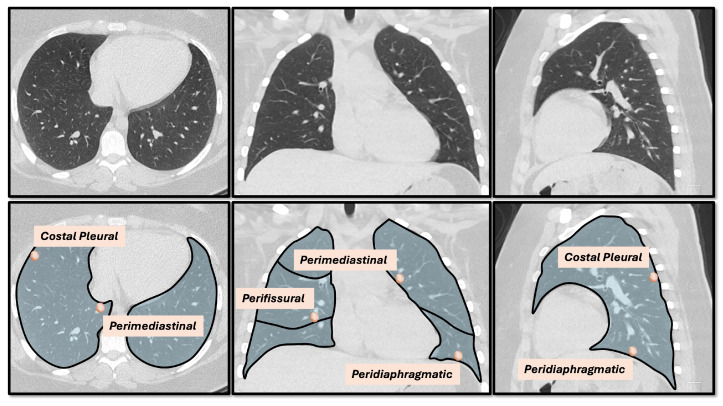
Schematic diagram of juxtapleural nodule subtypes. Schematic illustrating the anatomic classification of juxtapleural nodules, including perifissural, costal pleural, perimediastinal, and peridiaphragmatic locations. This schematic was created by the authors; classification terminology was informed by Lung-RADS v2022.

Juxtapleural nodules were categorized based on their reported anatomic relationship to pleural surfaces using the American College of Radiology (ACR) Lung-RADS v2022 [7] and the 2017 Fleischner Society Guidelines [4] (Figure 2). Nodules were classified into the following groups: juxtapleural/subpleural, perifissural, costal pleural, perimediastinal, and peridiaphragmatic [4,7]. Classification was based on terminology used in the radiology report and confirmed by imaging review when required.

For patients in the report-level concern cohort, available nodule descriptors were extracted from the original radiology reports, including reported anatomic location, nodule subtype, size when provided, interval change, attenuation/density when described, and morphologic features such as smooth, irregular, spiculated, pleural-based, perifissural, or fissural morphology. Because this study was designed as a retrospective report-level analysis rather than a standardized image re-review study, not all reports contained complete nodule-level descriptors. Therefore, imaging characteristics were summarized descriptively when available, and the absence of a reported descriptor was treated as missing rather than assumed negative.

### 2.3. Statistical Analysis

Analyses were conducted using GraphPad Prism (v11.0.0, GraphPad Software, Boston, MA, USA) and Orange Data Mining (v3.39.0, University of Ljubljana, Ljubljana, Slovenia) [12]. When assessing two continuous variables, either a two-sided Student’s *t*-test (for normally distributed data) or a Mann–Whitney U test (for non-Gaussian distributions) was used. When assessing two categorical variables, the Chi-square (χ^2^) test was used. To assess reproducibility of report-level classification, two independent readers reviewed the radiology reports meeting initial NLP-based eligibility criteria and classified each report according to whether it described a juxtapleural nodule as concerning for malignancy with a recommendation for follow-up imaging, PET/CT, biopsy, surgical evaluation, or other intervention. Inter-reader agreement was assessed using Cohen’s kappa with 95% confidence intervals. Discrepant cases were resolved by consensus review [13].

Two separate modeling analyses were performed. First, using the variables summarized in Table 1, a model was developed to classify whether a radiology report described a juxtapleural nodule considered concerning for malignancy with a recommendation for follow-up or intervention (N = 659). Candidate predictors included age group, sex, patient status, imaging modality, and health-system organization/site. Second, using the variables summarized in Table 2, a model was developed within the report-level concern cohort to classify whether the patient had a reported history of malignancy (N = 360). Candidate predictors similarly included age group, sex, patient status, imaging modality, and organization/site. For each analysis, a Naive Bayes classifier was used to generate a nomogram displaying the relative contribution of each variable to the predicted class probability. The nomogram was displayed using an odds-ratio scale to provide an interpretable estimate of the direction and magnitude of association for each feature. Logistic regression was then performed using 20-fold cross-validation with a class-balancing feature to evaluate discriminative performance. Model performance was summarized using receiver operating characteristic analysis, classification accuracy, sensitivity/recall, specificity, precision, F1 score, and confusion-matrix analysis. All results are reported as means with either standard deviation or percentages where appropriate, and statistical significance was defined as *p* ≤ 0.05.

## 3. Results

### 3.1. Cohort Identification

Between 1 January 2015 and 31 December 2024, 174,700 radiology reports contained terminology consistent with a juxtapleural nodule. After restricting to reports in which the impression raised concern for malignancy, 1196 patients were identified. Of these, 659 patients received a recommendation for imaging follow-up or intervention and were included in the final cohort (Figure 1).

### 3.2. Annual Recruitment Trends

The number of patients recruited per year increased over the study period (Figure S1), with the highest recruitment observed in the later study years. This trend paralleled increasing overall CT utilization and also likely reflects improved consistency in structured radiology reporting terminology through adopting best practices from Lung-RADS v2022 [7]. The number of examinations completed in 2020 was lower than in prior years, likely indicative of overall decreased radiological utilization secondary to the COVID-19 pandemic.

### 3.3. Demographics and Concern for Malignancy/Need for Follow-Up

Among the unique patients meeting the criteria for inclusion in the study (N = 85,435), 659 had reports describing a juxtapleural nodule considered concerning for malignancy with a recommendation for follow-up or intervention, whereas 84,776 encounters did not contain report-level language meeting these criteria; demographic characteristics are summarized in Table 1. Inter-reader agreement for classifying reports describing a juxtapleural nodule considered concerning for malignancy with a recommendation for follow-up or intervention was excellent, with a Cohen’s kappa of 0.93 [95% CI: 0.91–0.95]. Patients with reports describing a nodule considered concerning for malignancy by imaging criteria (N = 659) were older (68 vs. 64 years, *p* < 0.001), more likely to be seen in the inpatient setting (16% vs. 12%, *p* < 0.001), imaged more frequently with PET/CT (8% vs. 5%, *p* < 0.001), and were discovered on routine examinations (90% vs. 80%, *p* < 0.001), rather than an indication of STAT.

### 3.4. Predictive Modeling of Report-Level Concern for Malignancy

**Figure 3:**
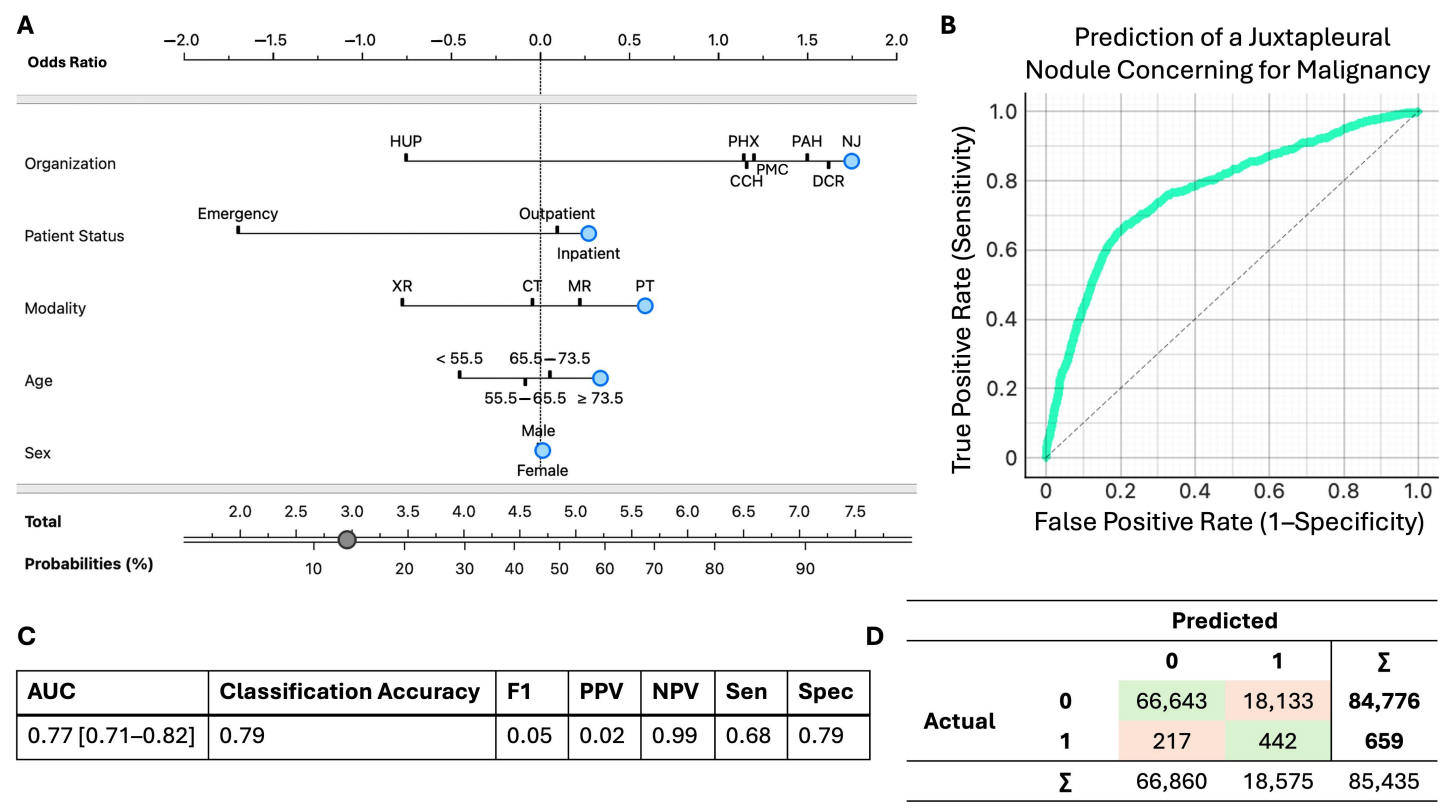
Predictive modeling of report-level concern for malignancy among patients with reported juxtapleural nodules. (**A**) Naive Bayes nomogram displaying odds-ratio-based feature contributions for classification of reports describing a juxtapleural nodule considered concerning for malignancy with follow-up or intervention recommendation. Candidate predictors included organization/site, patient status, imaging modality, age group, and sex. Features positioned farther from the null line contributed more strongly to the predicted class probability. (**B**) Receiver operating characteristic curve for logistic regression classification using 20-fold cross-validation. (**C**) Summary model performance metrics from cross-validation. (**D**) Confusion matrix for logistic regression classification. The numbers highlighted in green indicate a corect prediction whereas the numbers highlighted in red indicate an incorrect prediction. Organization sites included the main hospital sites (HUP = Hospital of the University of Pennsylvania, PMC = Presbyterian Medical Center) and community sites (PHX = Phoenixville Hospital, CCH = Chester County Hospital, PAH = Pennsylvania Hospital, DCR = Department of Community Radiology, NJ = Cherry Hill, New Jersey Hospital Systems).

Odds-ratio-based Naive Bayes nomogram and logistic regression model were generated to evaluate features associated with report-level concern for malignancy with follow-up or intervention recommendation. In the full cohort of 85,435 unique patient encounters, the nomogram demonstrated that organization/site, patient status, imaging modality, and age contributed more strongly to classification than sex (Figure 3A). Community sites, PET/CT imaging, older age, and non-emergency patient status were among the features associated with a higher predicted probability of report-level concern for malignancy, whereas sex contributed minimally to the model.

Using logistic regression with 20-fold cross-validation, the model predicted report-level concern for malignancy with follow-up or intervention recommendation (AUC: 0.77 [95% CI: 0.71–0.82]) (Figure 3B,C). Confusion-matrix analysis demonstrated 66,643 true-negative classifications, 442 true-positive classifications, 18,133 false-positive classifications, and 217 false-negative classifications, with a classification accuracy of 79% (Figure 3C,D).

### 3.5. Patients with a Juxtapleural Nodule Considered Concerning for Malignancy by Imaging Criteria

Within the subgroup of patients with a juxtapleural nodule undergoing follow-up (N = 659), 360 patients had a documented history of malignancy within the radiology report, and 299 had no known prior cancer diagnosis, per the radiology report (Table 2). Patients with a prior history of malignancy were most likely to have a new juxtapleural nodule considered concerning for malignancy by imaging criteria discovered in the outpatient setting (70% vs. 43%, *p* < 0.001), imaged more frequently with PET/CT (11% vs. 5%, *p* = 0.004), and were discovered on routine examinations (94% vs. 86%, *p* < 0.001), rather than an indication of STAT. Among patients with a previously reported history of malignancy (N = 360), the most common primary malignancies included non-small cell lung cancer, breast cancer, colorectal cancer, renal cell carcinoma, and squamous cell carcinoma (predominantly head and neck) (Figure S2). Within this group (N = 360), 21 patients had two known primary malignancies, and two patients had at least three known primary malignancies. This distribution is provided descriptively to characterize the oncologic background of patients in the report-level concern cohort and should not be interpreted as estimating malignancy-type-specific risk, as cancer-type denominator data were not available for the entire 85,435-patient cohort.

### 3.6. Predictive Modeling of Reported Prior Malignancy Within the Report-Level Concern Cohort

**Figure 4:**
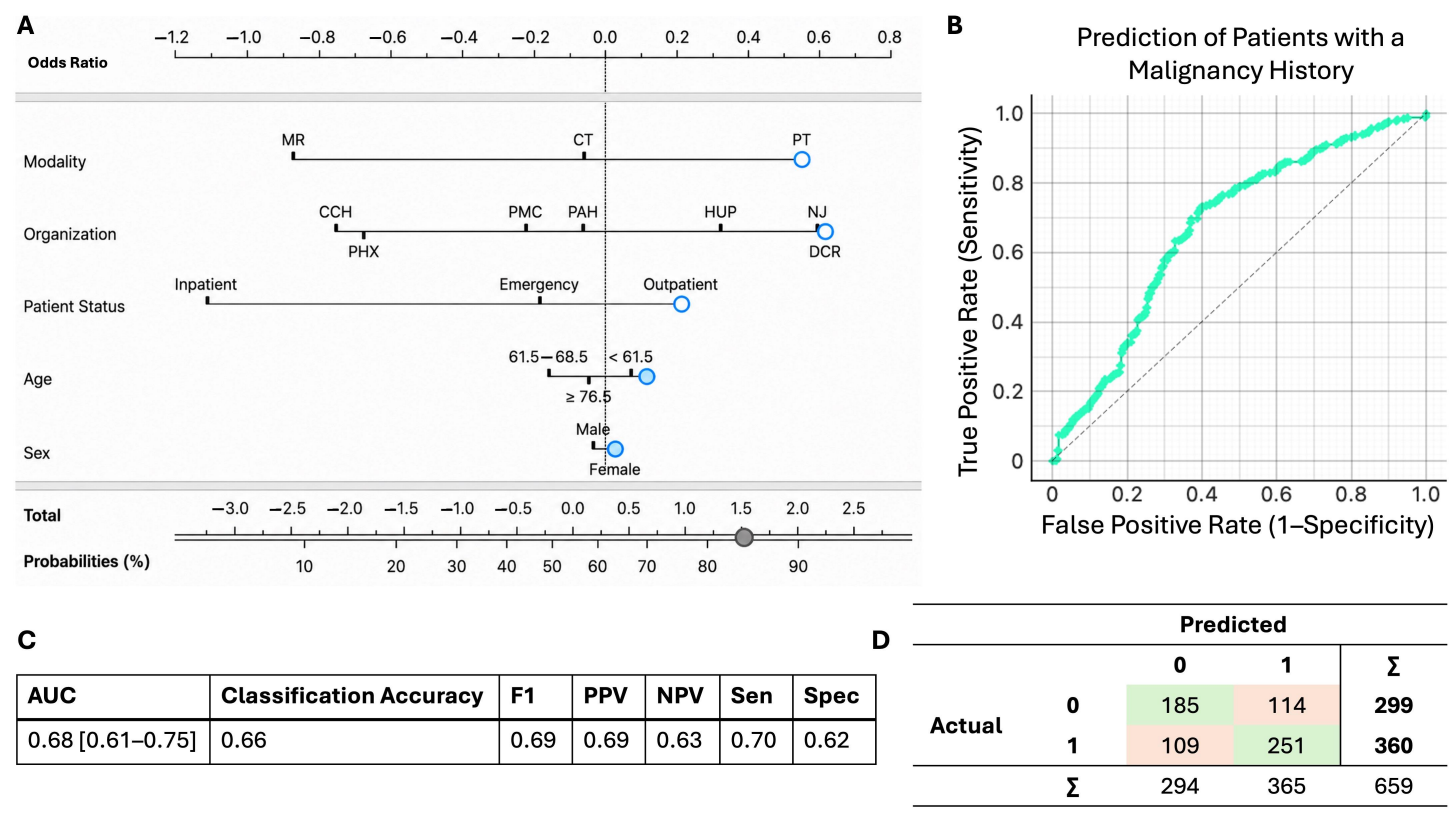
Predictive modeling of reported prior malignancy within the report-level concern cohort. (**A**) Naive Bayes nomogram displaying odds-ratio-based feature contributions for classification of reported prior malignancy among patients whose reports described a juxtapleural nodule considered concerning for malignancy with follow-up or intervention recommendation. Candidate predictors included organization/site, patient status, imaging modality, age group, and sex. Features positioned farther from the null line contributed more strongly to the predicted class probability. (**B**) Receiver operating characteristic curve for logistic regression classification using 20-fold cross-validation. (**C**) Summary model performance metrics from cross-validation. (**D**) Confusion matrix for logistic regression classification. The numbers highlighted in green indicate a corect prediction whereas the numbers highlighted in red indicate an incorrect prediction. Organization sites included the main hospital sites (HUP = Hospital of the University of Pennsylvania, PMC = Presbyterian Medical Center) and community sites (PHX = Phoenixville Hospital, CCH = Chester County Hospital, PAH = Pennsylvania Hospital, DCR = Department of Community Radiology, NJ = Cherry Hill, New Jersey Hospital Systems).

Odds-ratio-based Naive Bayes nomogram and logistic regression model were generated to evaluate features within the 659-patient report-level concern cohort to evaluate variables associated with reported prior malignancy (N = 360). The nomogram demonstrated that imaging modality, organization/site, and patient status contributed more strongly to classification than age or sex (Figure 4A). PET/CT imaging, community sites, and outpatient status were among the features associated with a higher predicted probability of patients with a reported history of malignancy, whereas sex contributed minimally to the model.

Using logistic regression with 20-fold cross-validation, the model predicted patients with a reported history of malignancy (AUC: 0.68 [95% CI: 0.61–0.75]) (Figure 4B,C). Confusion-matrix analysis demonstrated 185 true-negative classifications, 251 true-positive classifications, 114 false-positive classifications, and 109 false-negative classifications with a classification accuracy of 66% (Figure 4C,D).

### 3.7. Imaging Characteristics of Juxtapleural Nodules Prompting Malignancy-Directed Evaluation

**Figure 5:**
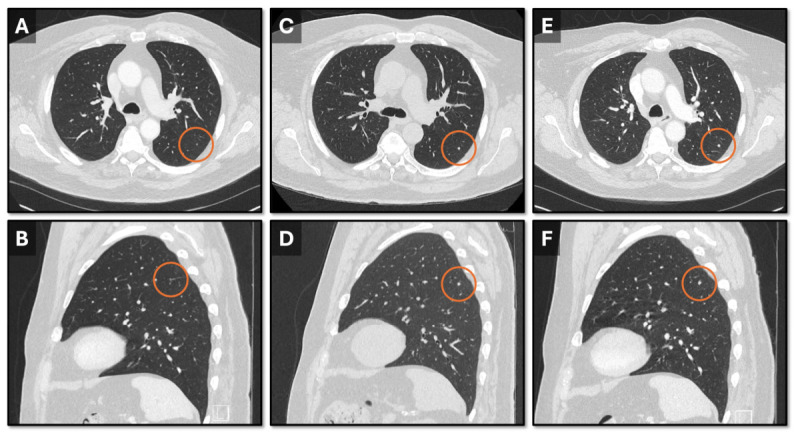
Left perifissural, biopsy-proven renal cell carcinoma (RCC). CT images at baseline in (**A**) axial and (**B**) coronal, 17 months from baseline in (**C**) axial and (**D**) coronal, and 23 months from baseline in (**E**) axial and (**F**) coronal. Nodule measures 2 mm at baseline, 4 mm at 17 months, and 6 mm at 23 months. The red circle highlights the concerning pulmonary nodule. Nodule was suspicious for malignancy based on interval growth and rounded morphology.

**Figure 6:**
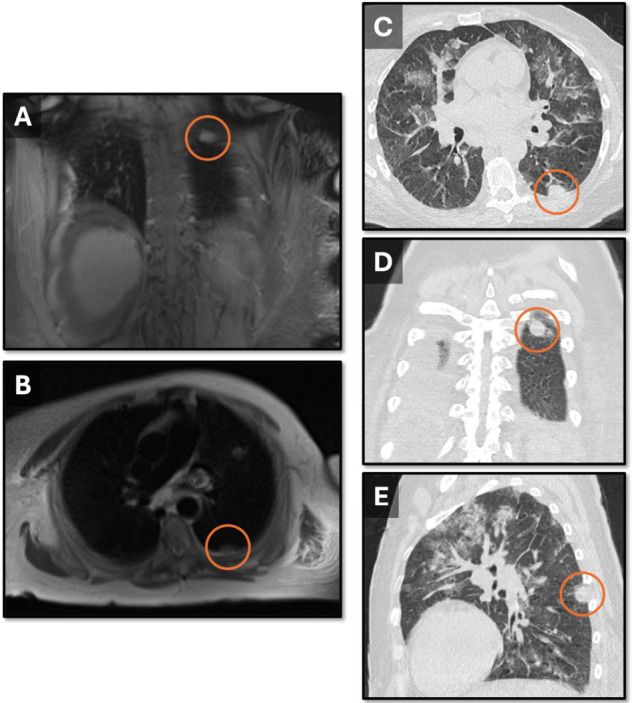
Left costal pleural nodule in a patient with a known primary of infiltrative hepatocellular carcinoma involving segment 8 of the liver with short segment tumor invasion of the proximal right portal vein. MR images at baseline in (**A**) coronal T1 3D Fat Saturated Post Contrast and (**B**) axial T2 Single-Shot Fast Spin Echo (SSFSE) and 5 days later on CT with (**C**) axial, (**D**) coronal, and (**E**) sagittal views. The red circle highlights the concerning pulmonary nodule. Nodule measures 14 mm in longest dimension at baseline and 5 days later. Nodule was suspicious for malignancy based on size.

**Figure 7:**
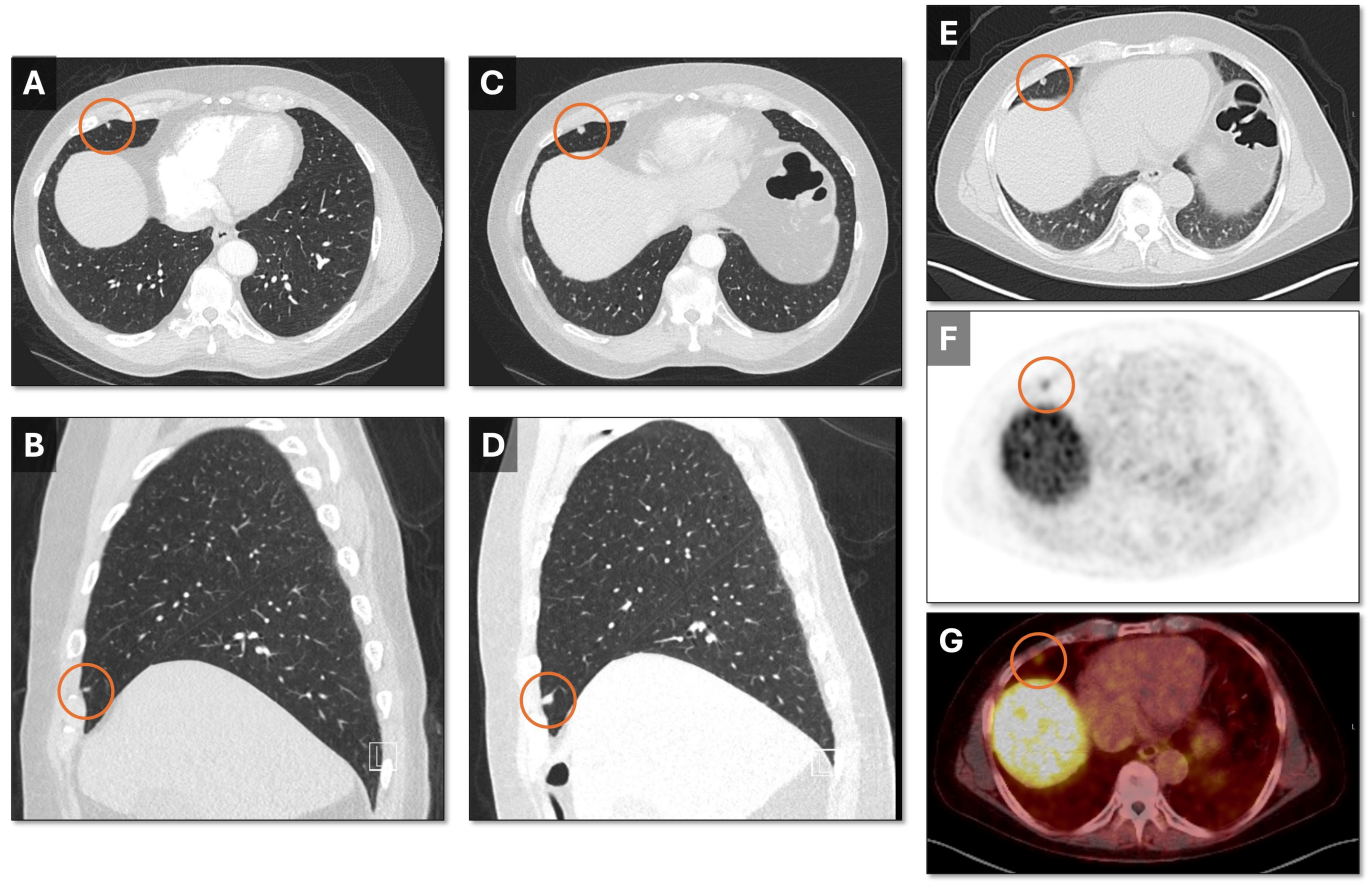
Empirically treated right middle lobe costal pleural nodule in a patient with a history of prostate cancer. CT images at baseline in (**A**) axial and (**B**) coronal, 5 years later from baseline in (**C**) axial and (**D**) coronal, and 5 years from baseline in axial (**E**) CT, (**F**) PSMA PET, and (**G**) PET/CT overlay. Nodule measures 2 mm at baseline, 5 mm at 5-year follow-up on both high-resolution chest CT (**C**,**D**) and PSMA PET/CT (**E**–**G**). The red circle highlights the concerning pulmonary nodule. Nodule was suspicious for malignancy based on interval growth and PSMA-avidity.

**Figure 8:**
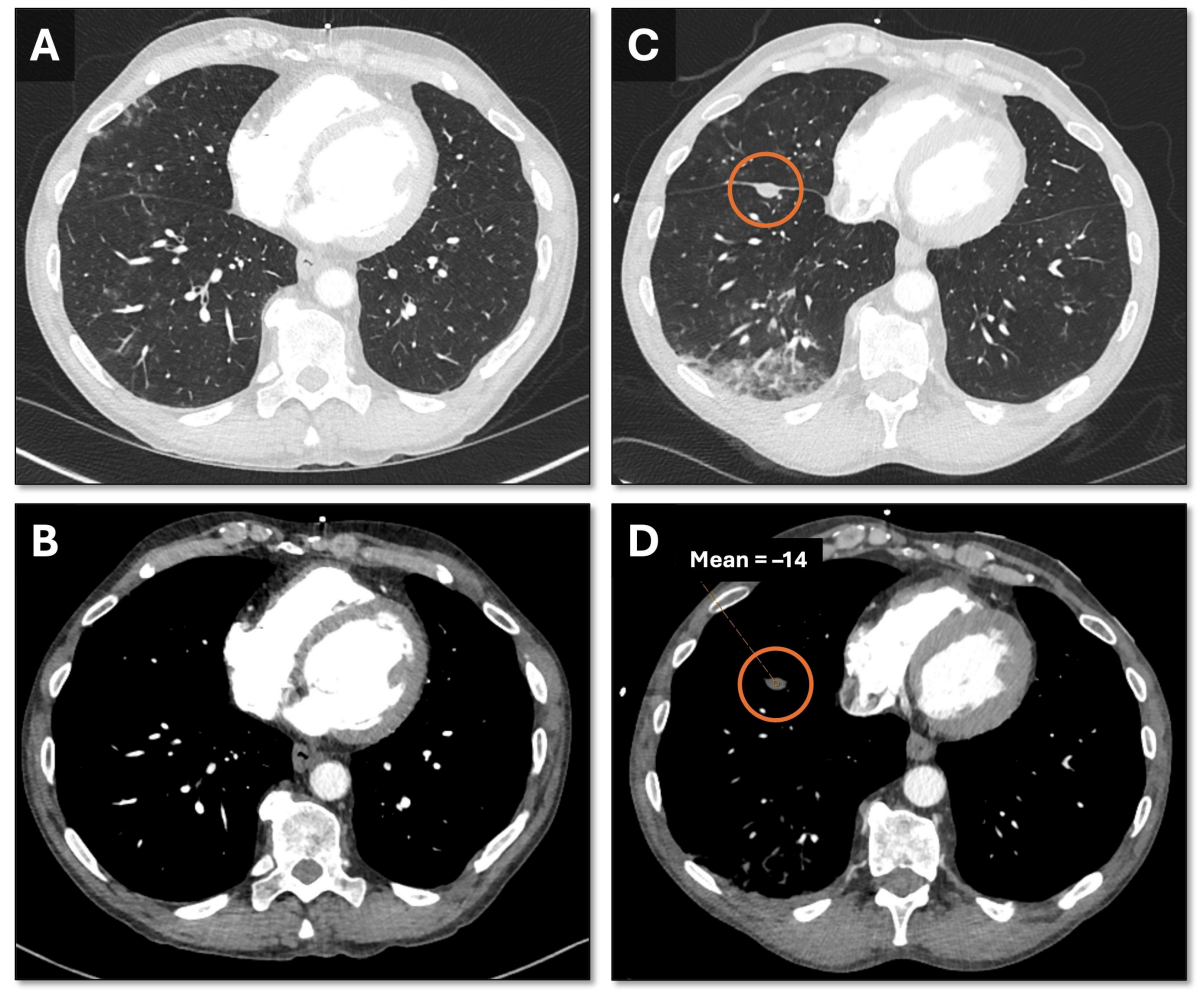
Loculated pleural effusion in a patient with a history of heart failure. An example of a benign mimic of juxtapleural nodules. Axial CT images prior to effusion in (**A**) lung and (**B**) mediastinal windows and after effusion in (**C**) lung and (**D**) mediastinal windows. The red circle highlights the loculated pleural effusion.The mean Hounsfield attenuation of the fluid is −14, based on delineated region of interest.

Representative cases of juxtapleural nodules that prompted malignancy-directed evaluation are provided as initially identified with CT (Figure 5), MRI (Figure 6), and PSMA PET/CT (Figure 7). Although most nodules were identified on CT or PET/CT, rare MRI or radiography cases were retained because this study evaluated report-level radiology concern and downstream management across modalities. These cases should not be interpreted as classic CT-defined Lung-RADS juxtapleural nodules, but rather as pleural-adjacent or subpleural lesions described using juxtapleural terminology in routine clinical reporting. We also provide some additional cases in the supplement for further characterization of malignant juxtapleural nodule transformation (Figures S3–S5). These cases mimicked benign perifissural nodules on initial assessment but were ultimately confirmed as metastatic disease or primary lung malignancy following biopsy or longitudinal progression. One example that can mimic malignant juxtapleural nodules includes intrafissural loculated effusions (Figure 8).

To evaluate how radiologists described pleural-adjacent nodules in routine reporting, we summarized the frequency of major anatomic descriptors used in the overall cohort and in the report-level concern cohort (Table 3). Generalized terminology such as “juxtapleural” or “subpleural” was used in the majority of reports in both cohorts, appearing in 76,050/84,776 reports (90%) in the overall cohort and 600/659 reports (91%) in the report-level concern cohort. More specific perifissural/juxtafissural terminology was used less frequently, appearing in 14,464/84,776 reports (17%) in the overall cohort and 113/659 reports (17%) in the report-level concern cohort. These categories were not mutually exclusive, as radiologists may use both broad and specific descriptors in the same report. Therefore, Table 3 should be interpreted as a summary of reporting terminology rather than an estimate of malignancy risk or follow-up/intervention rate by nodule subtype.

To better contextualize the report-level concern cohort, additional qualitative review of report-derived imaging descriptors was performed. Across the cohort, nodules prompting concern were most often described as juxtapleural/subpleural or costal pleural, with a smaller subset described as perifissural. Reported features contributing to concern included interval enlargement (*n* = 335), new nodule development (*n* = 183), atypical perifissural morphology (*n* = 54), pleural-based soft-tissue nodularity (*n* = 25), FDG avidity (*n* = 27), and concerning oncologic context. In contrast, typical benign-appearing perifissural nodules were generally described as small, smooth, solid, triangular, oval, or lentiform nodules adjacent to a fissure or pleural surface. Because size, attenuation, and margin descriptors were not uniformly present in all radiology reports, these characteristics were not analyzed as complete quantitative variables across the full cohort.

## 4. Discussion

This system-wide, retrospective analysis of 85,435 unique patient encounters represents one of the most comprehensive evaluations of juxtapleural nodules to date. Among these, only a small subset (659/85,435; <1%) were considered concerning for malignancy based on imaging criteria and clinical context, reinforcing the predominantly benign nature of these lesions in routine clinical practice. However, the presence of clinically meaningful malignant cases, particularly among patients with prior malignancy, highlights the importance of nuanced radiologic interpretation beyond simple categorization by nodule distribution.

Our findings align with and extend the framework established by Lung-RADS v2022, which incorporates juxtapleural nodules, including perifissural, costal pleural, perimediastinal, and peridiaphragmatic subtypes, into a structured lung cancer screening assessment. These guidelines emphasize that nodules meeting classic benign morphologic criteria (e.g., triangular, lentiform, or ovoid perifissural nodules < 10 mm) are overwhelmingly benign and typically represent intrapulmonary lymph nodes, with reported malignancy rates approaching 0% [7]. This concept has been corroborated by multiple screening cohorts, including analyses from the Dutch-Belgian NELSON trial and International Early Lung Cancer Action Program (I-ELCAP), which demonstrated that perifissural nodules rarely represent malignancy when strict morphologic criteria are applied [9,14,15].

The Fleischner Society guidelines similarly emphasize risk stratification based on size, growth, and clinical context, while acknowledging that morphology alone is insufficient for definitive characterization [4]. Nodule size and growth kinetics remain the most robust predictors of malignancy, with increasing diameter and shorter volume doubling times strongly associated with cancer risk [16]. However, reliance on size alone has limitations, as small nodules may still represent early malignancy, and benign nodules may demonstrate inter-observer and intra-observer measurement variability or grow subsequently due to superimposed inflammatory processes [16,17]. Our findings support this multifactorial paradigm: patients with nodules considered concerning for malignancy were significantly older, more frequently imaged with PET/CT, and more often evaluated in inpatient settings, suggesting that radiologists appropriately integrate clinical context with imaging features in risk stratification.

From an imaging perspective, several CT characteristics are critical in differentiating benign from malignant juxtapleural nodules. Benign perifissural nodules typically demonstrate smooth margins, homogeneous soft-tissue attenuation, and characteristic triangular, oval, or lentiform morphology abutting fissures or pleural surfaces [7]. In contrast, malignant nodules, whether primary lung cancers or metastases, more commonly exhibit irregular or spiculated margins, interval growth, heterogeneous attenuation, or deviation from typical perifissural morphology [11,18]. Importantly, our representative cases illustrate that malignant nodules may initially mimic benign perifissural nodules, particularly in early stages, underscoring the importance of longitudinal imaging and clinical correlation in patients with a prior history of malignancy, as they may have an atypical presentation [19].

An additional challenge in clinical practice is the presence of benign mimics of juxtapleural nodules. Loculated pleural effusions, focal pleural thickening, rounded atelectasis, and inflammatory or infectious processes may closely resemble true nodules on CT imaging. Intrafissural fluid collections, in particular, may mimic perifissural nodules but often demonstrate fluid attenuation, shape variability, or rapid interval change, aiding in differentiation. Recognition of these entities is essential to avoid unnecessary follow-up imaging or invasive diagnostic procedures.

Among the spectrum of juxtapleural nodules, perifissural nodules represent a distinct subclass that is most strongly associated with benign intrapulmonary lymph nodes. Histopathologic correlation has confirmed that these nodules mostly correspond to lymphatic tissue located along pulmonary fissures and pleural surfaces [6,9]. Their predictable morphology, stability over time, and lack of malignant potential have led to their classification as benign entities within both Lung-RADS and other guideline frameworks [7]. However, our findings suggest that when these nodules deviate from classic morphologic criteria, such as demonstrating atypical shape, interval growth, or occurring in patients with known malignancy, further evaluation may be warranted. This is particularly relevant in oncologic populations, where even nodules with benign-appearing morphology may represent metastatic disease [19,20].

The clinical implications of this study are highly relevant to both academic and community radiology practice. While structured reporting systems such as Lung-RADS provide essential standardization, real-world application requires careful integration of imaging features with patient-specific clinical factors. Over-reliance on morphology alone may result in under-recognition of malignant lesions, whereas excessive caution may lead to increased healthcare utilization, patient anxiety, and unnecessary procedures. Our findings support a balanced, risk-adapted approach that incorporates morphology, growth, and clinical context.

This study has several limitations. Most importantly, the study endpoint should not be interpreted as a definitive malignancy diagnosis. Because the cohort was identified using radiology report terminology and follow-up/intervention recommendations, the analysis captures radiologist concern and downstream management patterns rather than uniformly confirmed malignant outcomes. Pathology, resection, or standardized long-term imaging follow-up was not available for all cases, creating potential for both false-positive classification of benign lesions as suspicious and false-negative classification of malignant lesions that were not described using the queried terminology. Accordingly, the reported proportion of patients with concerning nodules should not be interpreted as the true malignancy rate of juxtapleural nodules. Because this is a retrospective NLP-based analysis, it relies on the accuracy and consistency of radiology report terminology, which may introduce classification bias. Additionally, variability in imaging technique and reporting practices across a 10-year period may influence results.

While this analysis demonstrated excellent reproducibility for report-level classification, it does not eliminate potential variability in the original clinical radiology interpretations. The study, therefore, evaluates real-world radiology reporting and management patterns rather than standardized independent image interpretation. Further, nodule-level imaging features, including size, attenuation, morphology, and margin characteristics, were extracted from original radiology reports and were not uniformly available for all patients. As a result, the study cannot provide a fully standardized image-level characterization of every nodule in the report-level concern cohort. Lastly, our predictive models were intended to provide effect-size and feature-importance context for the observed associations and should not be interpreted as validated clinical prediction tools. In particular, the full-cohort model was affected by substantial class imbalance because report-level concern for malignancy was rare, noting that we utilized class-balancing tools while performing the logistic regression. Future prospective studies incorporating standardized imaging review, volumetric analysis, and longitudinal outcome validation are warranted to further refine risk stratification models. Future work should focus on juxtafissural nodules of <10 mm, as proposed in Lung-RADS 2022, and investigate their potential malignancy risk using a standardized review of typical and atypical CT imaging features.

## 5. Conclusions

In conclusion, although the majority of juxtapleural nodules are benign, a clinically significant subset, particularly in patients with prior malignancy, may represent malignant disease. Accurate differentiation requires integration of morphologic features, growth patterns, and clinical context. These findings support and extend current guideline-based frameworks, emphasizing the importance of careful evaluation of atypical or evolving juxtapleural nodules to optimize patient management.

## Figures and Tables

**Table 1:** Demographics of patients with a juxtapleural nodule considered concerning or not concerning for malignancy by imaging criteria. Comparison of demographic and imaging characteristics between patients with a juxtapleural nodule considered concerning (N = 659) or not concerning (N = 84,776) for malignancy by imaging criteria. Organization sites included the main hospital sites (HUP = Hospital of the University of Pennsylvania, PMC = Presbyterian Medical Center) and community sites (PHX = Phoenixville Hospital, CCH = Chester County Hospital, PAH = Pennsylvania Hospital, DCR = Department of Community Radiology, NJ = Cherry Hill, New Jersey Hospital Systems).

Demographics of Patients with a Juxtapleural Nodule Considered Concerning or Not Concerning for Malignancy by Imaging Criteria			
Parameter	Concerning for Malignancy (N = 659)	Not Concerning for Malignancy (N = 84,776)	*p*-Value
Age (years)	67.7 ± 12.9	63.6 ± 14.8	<0.001
Sex (% Female)	54% (356)	53% (45,280)	0.95
Patient Status			<0.001
Emergency Department	2% (15)	13% (11,218)	
Inpatient	16% (103)	12% (10,189)	
Outpatient	58% (381)	66% (55,882)	
Not Specified	24% (160)	9% (7487)	
Modality			<0.001
Computed Tomography (CT)	92% (603)	95% (80,421)	
Magnetic Resonance Imaging (MRI)	0.2% (1)	0.2% (205)	
Nuclear Medicine (NM)/Positron emission tomography (PET)	8% (55)	5% (3880)	
Radiography	0.0% (0)	0.3% (278)	
Organization			<0.001
Main Hospitals			
HUP	258 (39%)	70,760 (84%)	
PMC	49 (7%)	1936 (2%)	
Community Hospitals			
PHX	122 (19%)	5044 (6%)	
CCH	68 (10%)	2784 (3%)	
PAH	47 (7%)	1378 (1%)	
DCR	82 (12%)	2114 (2%)	
NJ	33 (5%)	760 (1%)	

**Table 2:** Demographics of patients with a juxtapleural nodule considered concerning for malignancy by imaging criteria with or without a prior history of malignancy. Comparison of demographic and imaging characteristics among patients with juxtapleural nodules considered concerning for malignancy, stratified by presence (N = 360) or absence (N = 299) of prior reported malignancy. Organization sites included the main hospital sites (HUP = Hospital of the University of Pennsylvania, PMC = Presbyterian Medical Center) and community sites (PHX = Phoenixville Hospital, CCH = Chester County Hospital, PAH = Pennsylvania Hospital, DCR = Department of Community Radiology, NJ = Cherry Hill, New Jersey Hospital Systems).

Demographics of Patients with a Juxtapleural Nodule Considered Concerning for Malignancy by Imaging Criteria with or Without a Prior History of Malignancy			
Parameter	Reported History of Malignancy (N = 360)	No Reported History of Malignancy (N = 299)	*p*-Value
Age (years)	67.5 ± 13.1	68.1 ± 12.7	0.57
Sex (% Female)	55% (197)	53% (159)	0.75
Patient Status			<0.001
Emergency Department	2% (7)	2% (7)	
Inpatient	8% (29)	25% (75)	
Outpatient	70% (253)	43% (128)	
Not Specified	20% (71)	30% (89)	
Modality			0.004
Computed Tomography (CT)	89% (321)	94% (282)	
Magnetic Resonance Imaging (MRI)	0% (0)	0.3% (1)	
Nuclear Medicine (NM)/Positron emission tomography (PET)	11% (39)	5% (16)	
Radiography	0% (0)	0% (0)	
Organization			<0.001
Main Hospitals			
HUP	160 (44%)	96 (32%)	
PMC	24 (7%)	25 (8%)	
Community Hospitals			
PHX	46 (13%)	76 (25%)	
CCH	25 (7%)	45 (15%)	
PAH	25 (7%)	22 (7%)	
DCR	57 (16%)	25 (8%)	
NJ	23 (6%)	10 (3%)	

**Table 3:** Reported terminology used to describe juxtapleural nodule location. Frequency of major anatomic descriptors used in radiology reports among the overall cohort and the report-level concern cohort. Percentages are calculated within each cohort column. Categories are based on report terminology and are not mutually exclusive; a single report could contain more than one anatomic descriptor. Percentages are calculated within each cohort column and should not be interpreted as follow-up/intervention rates or malignancy risk by nodule subtype.

Reported Terminology Used to Describe Juxtapleural Nodule Location		
Reported Nodule Location Term	Overall Cohort: Reports Using Term, *n*/N (%)	Report-Level Concern Cohort: Reports Using Term, *n*/N (%)
Juxtapleural/subpleural	76,050/84,776 (90%)	600/659 (91%)
Perifissural/juxtafissural	14,464/84,776 (17%)	113/659 (17%)

## Data Availability

The data presented in this study are available on request from the corresponding author due to institutional and privacy restrictions.

## Supplementary Materials

The following supporting information can be downloaded at: https://www.mdpi.com/article/10.3390/diagnostics16111663/s1, Figure S1. Patient Recruitment Over Time. Figure S2. Distribution of Primary Malignancies. Figure S3. Left costal pleural biopsy-proven intrahepatic cholangiocarcinoma metastasis. Figure S4. Left costal pleural biopsy-proven adenocarcinoma, suspected metastatic secondary to right upper lobe adenocarcinoma status post right upper lobectomy and right lower lobe segmentectomy. Figure S5. Right costal pleural nodule in a patient with a history of metastatic colon cancer.
