# Supplementary material for: A 10-Year Comprehensive, Single-Center, Retrospective Analysis on Juxtapleural Nodules: Insights into Classification and Risk
Source: Diagnostics (Basel). 2026 May 28;16(11):1663. doi: 10.3390/diagnostics16111663 (PMC13257318; doi:10.3390/diagnostics16111663)
Supplement: Supplementary file 1 [file diagnostics-16-01663-s001.zip › diagnostics-4313206-supplementary.pdf]

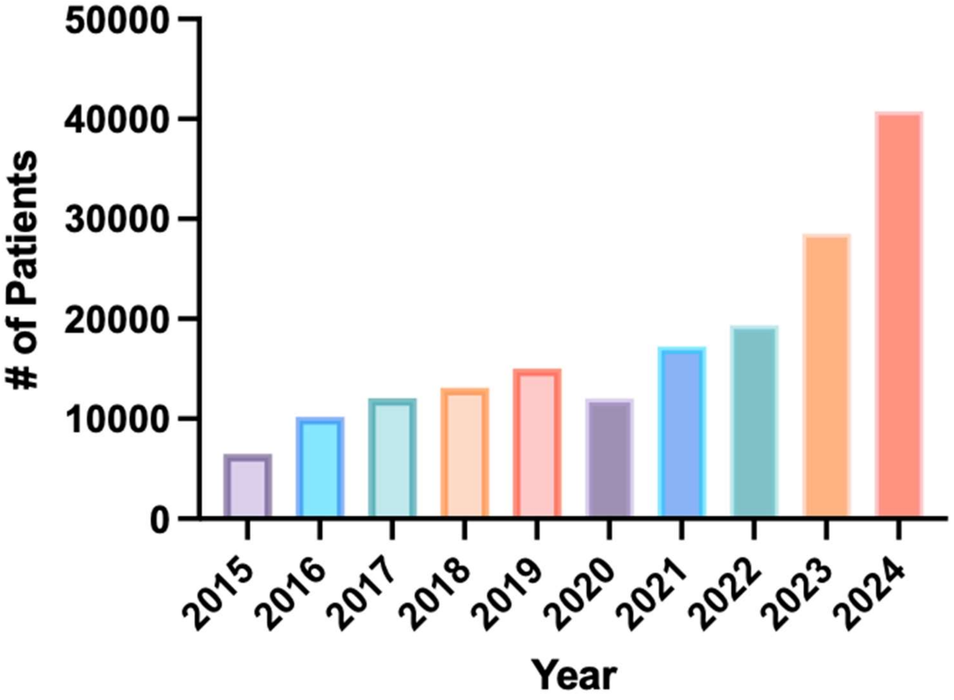

Figure S1. Patient Recruitment Over Time. Annual distribution of patients included in the initial recruitment (174,700), demonstrating increasing case identification over the study period, with a transient decrease in 2020 corresponding to reduced imaging utilization during the COVID-19 pandemic.

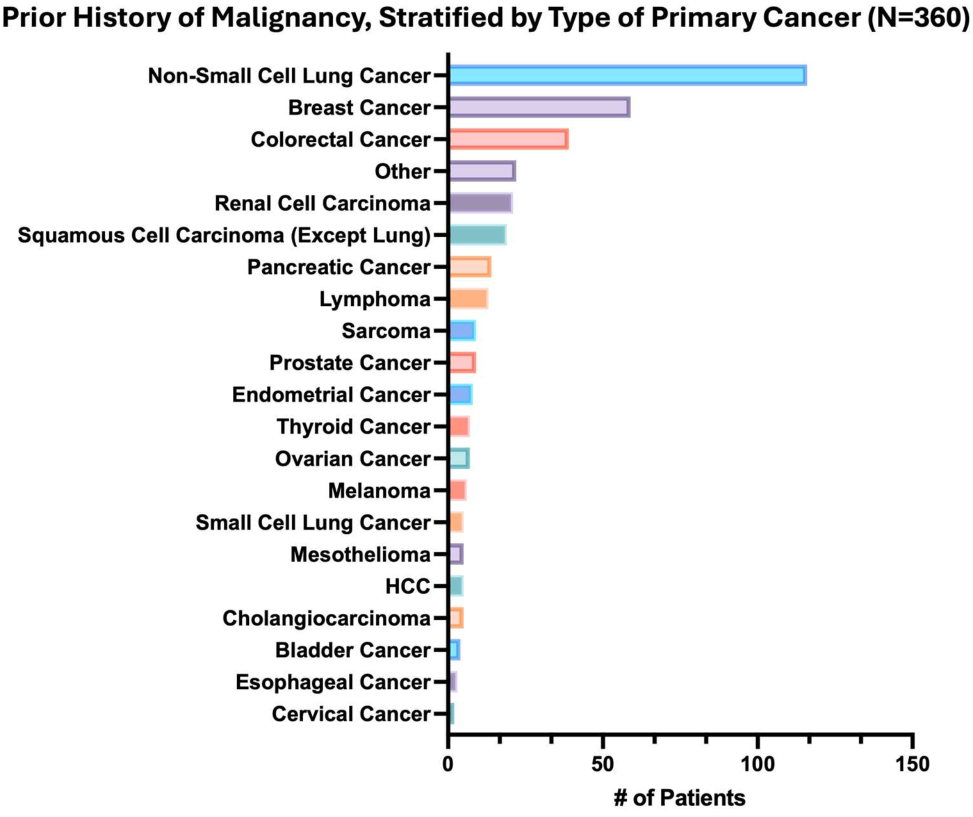

Figure S2. Distribution of Primary Malignancies. Breakdown of primary malignancies among patients with a reported history of cancer (N=360),

highlighting the most common tumor types associated with juxtapleural nodules concerning for malignancy by imaging criteria. This figure is intended as a descriptive summary of malignancy types among patients whose reports described a juxtapleural nodule as concerning for malignancy with follow-up or intervention recommendation. It does not estimate malignancy-type-specific risk because cancer-type denominator data were not available for the full cohort of 85,435 unique encounters.

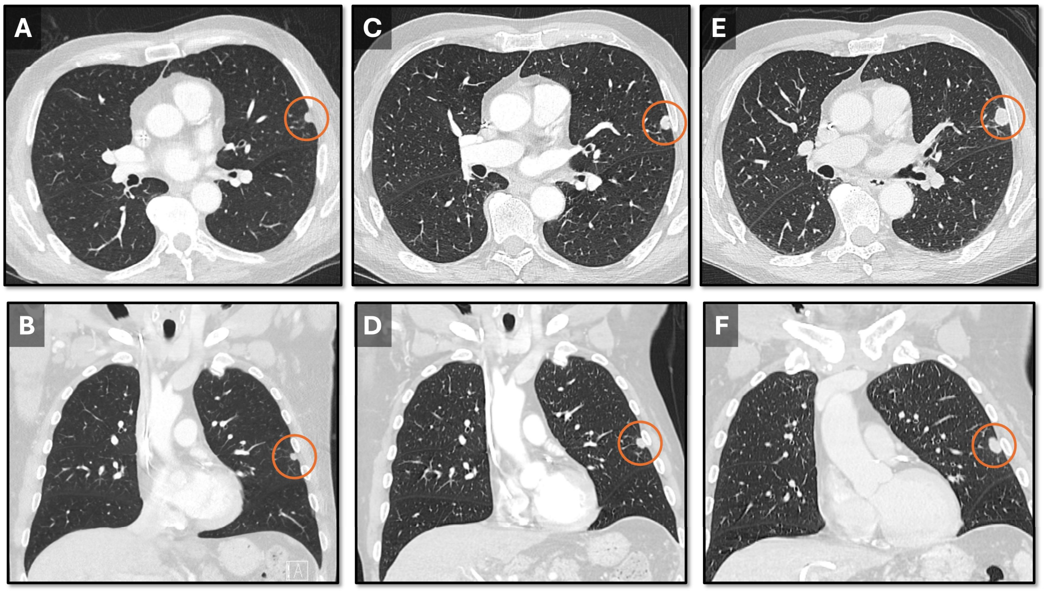

Figure S3. Left costal pleural biopsy-proven intrahepatic cholangiocarcinoma metastasis. CT images at baseline in (A) axial and (B) coronal, 10 months from baseline in (C) axial and (D) coronal, and 13 months from baseline in (E) axial and (F) coronal. The nodule measured 4 mm at baseline, 7 mm at 10 months, and 10 mm at 13 months, measured in short axis. The red circle highlights the concerning pulmonary nodule. Nodule was suspicious for malignancy based on interval growth and rounded morphology.

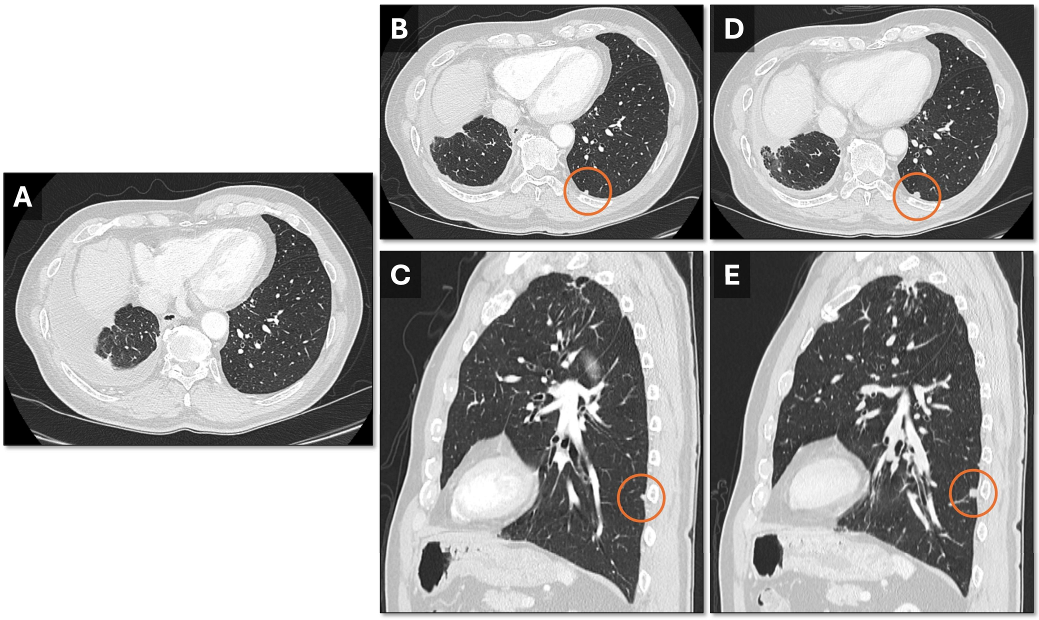

Figure S4. Left costal pleural biopsy-proven adenocarcinoma, suspected metastatic secondary to right upper lobe adenocarcinoma status post right

upper lobectomy and right lower lobe segmentectomy. Axial CT image at (A) baseline, (B) axial and (C) sagittal at 2 years from baseline, and (D) axial and (E) sagittal 5 years from baseline. Nodule is not present at baseline, 4 mm at 2 years from baseline, and 6 mm at 5 years from baseline, in short axis. The red circle highlights the concerning pulmonary nodule. Nodule was suspicious for malignancy based on interval growth.

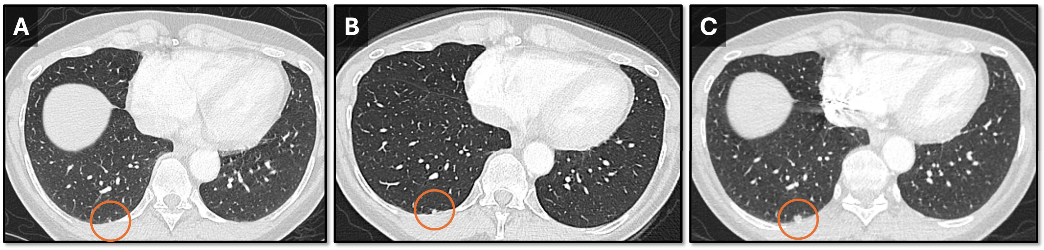

Figure S5. Right costal pleural nodule in a patient with a history of metastatic colon cancer. Axial CT images at (A) baseline, (B) 2 months from baseline, and (C) 9 months from baseline. The red circle highlights the concerning pulmonary nodule. Nodule is not present at baseline, 5 mm at 2 months, and 13 mm at 9 months, in short axis.
